# Polysialic Acid in Human Plasma Can Compensate the Cytotoxicity of Histones

**DOI:** 10.3390/ijms19061679

**Published:** 2018-06-05

**Authors:** Kristina Zlatina, Max Saftenberger, Andrea Kühnle, Christina E. Galuska, Ulrich Gärtner, Alexander Rebl, Michael Oster, Andreas Vernunft, Sebastian P. Galuska

**Affiliations:** 1 Institute of Reproductive Biology, Leibniz Institute for Farm Animal Biology (FBN), Wilhelm-Stahl-Allee 2, 18196 Dummerstorf, Germany; zlatina@fbn-dummerstorf.de (K.Z.); kuehnle@fbn-dummerstorf.de (A.K.); galuska.christina@fbn-dummerstorf.de (C.E.G.); rebl@fbn-dummerstorf.de (A.R.); oster@fbn-dummerstorf.de (M.O.); vernunft@fbn-dummerstorf.de (A.V.); 2 Institute of Biochemistry, Faculty of Medicine, Justus-Liebig-University, Friedrichstr. 24, 35392 Giessen, Germany; max.saftenberger@gmx.de; 3 Institute of Anatomy and Cell Biology, Justus-Liebig-University, Aulweg 123, 35385 Giessen, Germany; ulrich.gaertner@anatomie.med.uni-giessen.de

**Keywords:** polysialic acid, plasma, histone, neutrophil extracellular traps, NETs

## Abstract

The innate immune system has numerous mechanisms to fight against pathogens, including the formation of neutrophil extracellular traps (NETs). By spreading out chromatin, antimicrobial peptides and enzymes, neutrophils efficiently trap pathogens like bacteria and facilitate their elimination. During this process, high concentrations of extracellular histones can be reached. Several researchers have demonstrated that the cytotoxic characteristics of these histones can trigger diseases like sepsis. Interestingly, the carbohydrate polysialic acid (polySia) can bind histones and reduce histone-mediated cytotoxicity in a chain length-dependent manner. In the present study, we examined the chain length of polySia in plasma and tested its ability to decrease the cytotoxic characteristics of extracellular histones. Remarkably, we detected polySia not only in the soluble fraction of plasma, but also on enriched extracellular vesicles (EVs). Chain length analysis revealed that polySia chains originating from human plasma can consists of more than 40 sialic acid residues and show a cytoprotective effect against extracellular histones. Intriguingly, polySia is not only present in human plasma but also in fish and other branches of vertebrates. Thus, polySia is a physiological element in plasma and may represent a natural buffer for extracellular histones.

## 1. Introduction

Polysialic acid (polySia) is a negatively charged carbohydrate consisting of α2,8-linked *N*-acetylneuraminic acid residues in mammalians [1]. Two polysialyltransferases can modify distinct proteins, e.g., the neural cell adhesion molecule (NCAM), with this posttranslational modification [2]. Besides being involved in the plasticity of the nervous system [3,4,5] and the development of several organs [6], polySia plays also a role in immunological processes [2]. For instance, polySia is present on activated dendritic cells, where it has been described as a regulator of cell trafficking [7,8]. Furthermore, polySia has been discussed as a potential tool to reduce the negative outcomes of neutrophil extracellular traps (NETs). Usually, neutrophils use NETosis as a mechanism to fight pathogens [9,10]. For that purpose, NETs consist of enzymes and antimicrobial peptides that are attached to the released DNA [11]. However, in addition, these DNA-meshworks contain high concentrations of extracellular histones [12], which are toxic for endogenous cells, thus, triggering, for example, autoimmune diseases, sepsis and infertility [13,14,15,16]. Therefore, it is desirable to limit excessive NET formation and/or to deactivate the cytotoxicity of extracellular histones with components like polySia. Comparable effects against the cytotoxicity of histones could be observed with heparin [17].

Recently, a more detailed examination showed how polySia may interact with the individual histones H1, H2A, H2B, H3 and H4 [18]. The study demonstrated that all histones can be bound and inactivated in a chain length-dependent manner. In addition to free polySia and polysialylated glycoconjugates, polySia attached to, e.g., latex beads can be cytoprotective against histones [19]. Based on these results, we speculated that polySia on cellular membranes and as soluble components in body fluids may represent a natural “buffer system” for extracellular histones [6,15,18,19,20,21].

Since NETosis occurs frequently in the blood stream and studies have already shown the presence of polySia in blood [22,23,24], we examined polySia in human plasma samples for its capability to counteract the cytotoxicity of histones. The outlined experiments demonstrated that polySia originating from human plasma can consist of more than 40 sialic acid residues and is able to be cytoprotective against extracellular histones. Interestingly, polySia is not only present in human plasma but also in different branches of vertebrates including bony fish.

## 2. Results and Discussion

### 2.1. Plasma Contains Polysialic Acid

Several studies have shown that polySia can be present in human plasma as well as serum samples and that the polySia level can be altered during diseases like schizophrenia and in childhood neuroblastoma [22,23,24]. These researchers mainly tested polySia for a potential biomarker for certain neuropathological changes. We were interested whether polySia in plasma from healthy donors has the structural requirements to modulate the cytotoxicity of extracellular histones. For these experiments, we used commercially available pooled plasma. We choose this with the intent to ensure reproducible results that would be independent of potential variations of individual donors’ polySia.

**Figure 1:**
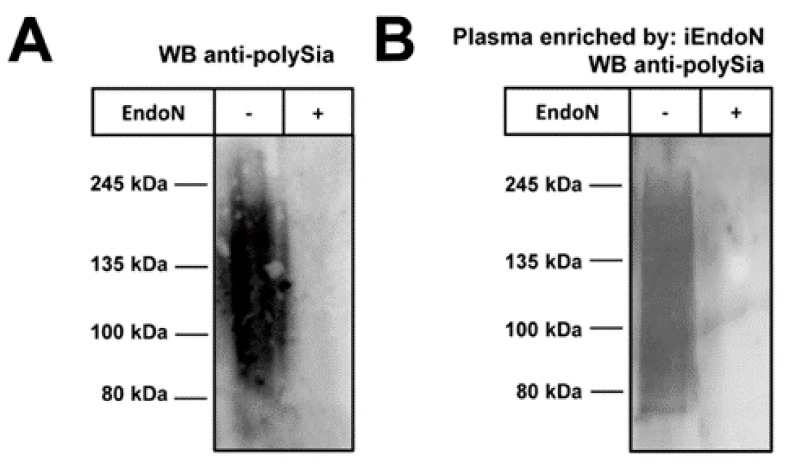
PolySia occurs in human plasma: (**A**) Pooled plasma samples were used for Western blots to detect polySia. A specific binding of mAb 735 was confirmed by a pretreatment of plasma with endoN; and (**B**) Polysialylated carriers were isolated from plasma using inactive endoN coupled to magnetic beads and analyzed by Western blots, as depicted in (**A**).

Initially, we tested the plasma pool for polySia. To this end, Western blots against polySia were performed using the mAb 735 which showed the typical smear of polysialylated proteins (Figure 1A).

A degradation of polySia by endoneuraminidase (endoN), however, abolished the immunostaining, thus verifying the specificity of the signal.

Next, polySia was isolated from plasma via magnetic beads coated with inactive endoN. The inactive form of endoN can bind polySia, but is unable to degrade the polymer [25]. In line with the plasma samples, we observed the characteristic smear of the eluted polySia-fraction by Western blotting (Figure 1B).

### 2.2. Extacellular Vesicles Can Contain PolySia

In addition to soluble biomolecules, plasma contains extracellular vesicles (EVs). EVs are small structures enclosed by lipid-bilayer released by all tested cell types as known so far [26]. They are grouped depending on their release mechanism, including: exosomes released by exocytosis; microvesicles (also called ectosomes, shedding microvesicles or microparticles) resulting from the budding of plasma membranes; and apoptotic vesicles/bodies budding from the plasma membrane during apoptosis. Besides the non-unified nomenclature, their sizes are also very heterogeneous. Depending on the sources, sizes may range: in the case of exosomes, from 40–100 nm; microvesicles range 20–1000 nm; and apoptotic vesicles range 50–5000 nm [26,27,28]. Inside and embedded in the lipid bilayer, EVs carry proteins, including enzymes and receptors, different types of nucleotides, carbohydrates, further lipids and metabolites [26]. In general, EVs represent an efficient and useful communication tool between cells, which seems to be conserved because even bacteria use EVs for communication with each other [28].

**Figure 2:**
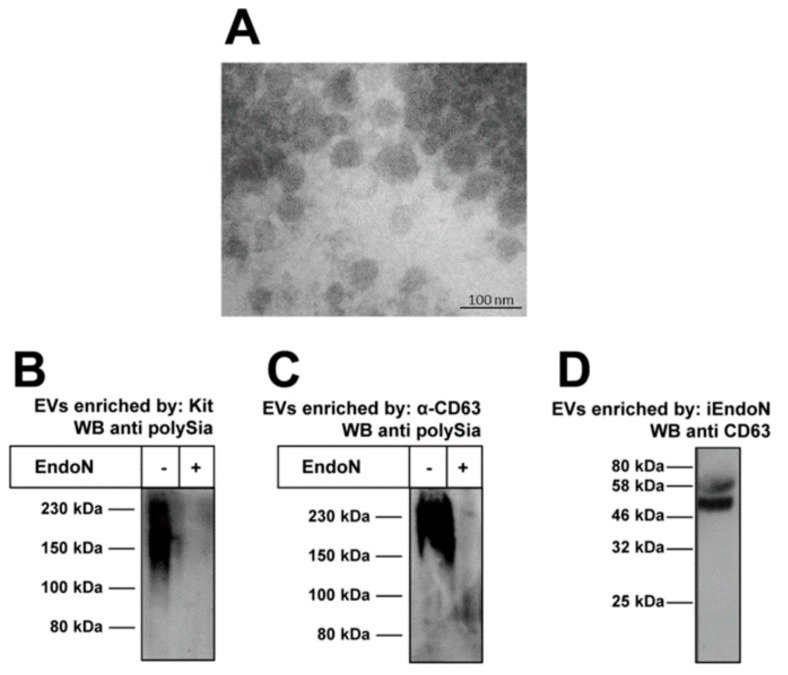
Extracellular vesicles (EVs) can be polySia positive. (**A**) TEM revealed that enriched EVs mainly have diameters of ~100 nm; (**B**) EVs were isolated using a centrifugation kit and enriched fractions were tested for polySia by Western blotting. As a negative control, polySia was degraded by endoN; (**C**) In addition, EVs were isolated by magnetic beads coated with an antibody against CD63 and polySia was visualized; and (**D**) PolySia containing samples were isolated by inactive endoN (iEndoN) and checked for the presence of the EV marker protein CD63.

We wanted to test whether EVs in plasma contain polySia. For this purpose, we enriched EVs from plasma using commercially available centrifugation kits. The enriched fractions were analyzed by transmission electron microscopy (TEM) (Figure 2A), visualizing mainly vesicles with diameters of ~100 nm.

In addition, Western blots against polySia were performed. As shown in Figure 2B, polySia staining was detected using analogously enriched EV-fractions. A degradation of polySia by endoN prevented immunostaining. Similar results were obtained when EVs were enriched more specifically using magnetic beads coated with a mAb against the EV marker protein CD63 (Figure 2C).

Moreover, an inverse application was performed to confirm the presence of polySia on EVs. For this purpose, we again used magnetic beads coated with inactive endoN for isolation. The eluted samples were checked for the presence of the EV marker protein CD63 to investigate whether EVs were purified in parallel. CD63 was detected by Western blotting (Figure 2D). Thus, EVs seem to be isolated due to polySia on their surface. Based on our recent study showing that polysialylated fluorescence beads accumulate via polySia on NET-fibers [19], it might be possible that EVs can also attach to NET, if the degree of polymerization of free segments and the density of polySia-chains on EVs is high enough to stabilize an attachment in blood stream. However, until now no unambiguous evidence exists for this possibility and, if e.g., the polysialylated glycoconjugates are permanently integrated into the membrane of EVs (integral membrane glycoproteins) or only associated (peripheral membrane glycoproteins).

### 2.3. Chain Length Determination of PolySia from Plasma

**Figure 3:**
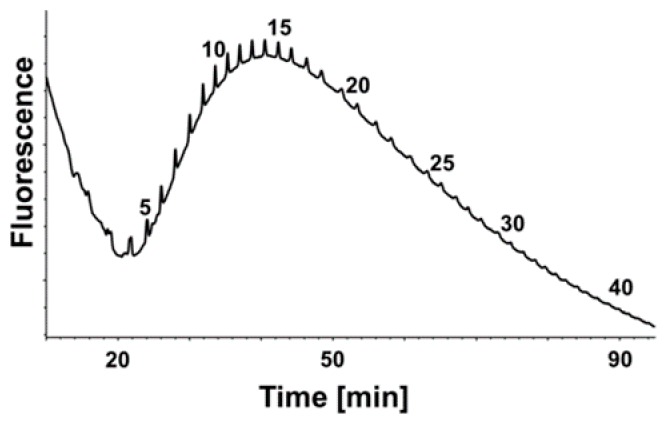
PolySia occurs in various degrees of polymerization (DP) in human plasma. The DP was analyzed by anion exchange HPLC after DMB-labeling, and the chain lengths are given for selected peaks on top of the profiles. The obtained samples were enriched by magnetic beads coated with inactive endoN from plasma.

As recently shown, polySia chains must consist of more than 20 sialic acid residues to bind histones, as well as to neutralize their cytotoxicity [6,19]. In order to test if polySia from human plasma is capable of interacting with histones, we analyzed the degree of polymerization (DP) using the enriched polySia-fraction. Therefore, polySia was fluorescently labeled with 4,5-methylene dioxybenzene (DMB) and separated by HPLC using an anion exchange column. The obtained chromatograms displayed polySia chains up to lengths of more than 40 sialic acid residues (Figure 3). Thus, the chain length analysis suggests that the binding to and deactivation of histones should be possible using polySia from plasma.

### 2.4. Histone H1 Is a Natural Binding Partner of PolySia in Human Plasma

Interestingly, extracellular histones circulate in the blood of healthy individuals at a concentration of ~0.06 ng/mL (serum samples). This concentration can dramatically increase up to 3 ng/mL after trauma [29]. In this study, we investigated if histones in plasma are also natural binding partners of polySia.

**Figure 4:**
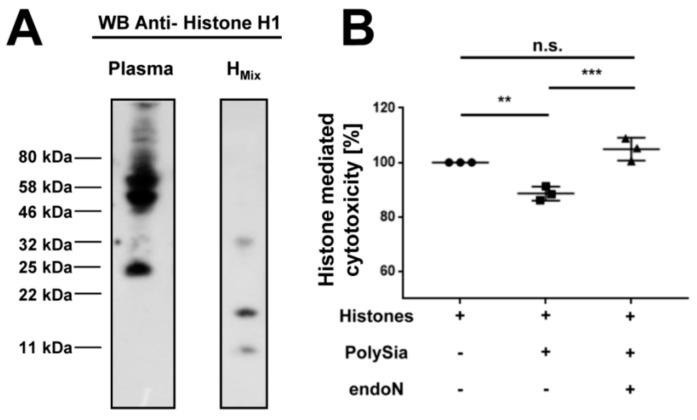
PolySia originating from plasma is cytoprotective, despite histone H1 as binding partner. (**A**) In order to test if polySia interacts with histones in plasma, Western blotting against histone H1 was performed. PolySia purification was done by inactive endoN from plasma. A commercially available histone mixture from calf thymus served as a positive control; and (**B**) The impact of plasma polySia was analyzed. To this end, cells were treated with histones (40 µg/mL) and the cytotoxicity was determined. In parallel, the cytotoxicity was analyzed in the presence of polySia isolated from plasma. In addition, the polySia fractions were previously treated with active endoN. 100% cytotoxicity was set for histone-treated cells. All values are means of 3 independent experiments. The statistical evaluation was performed by Two-Way ANOVA analysis including Tukey’s multiple comparison test. n.s., not significant; ** *p* < 0.01; *** *p* < 0.001. black dot: histones +, polySia -, endoN -; square: histones +, polySia +, endoN -; triangle: histones +, polySia +, endoN +.

To achieve this, we purified the polySia from plasma by inactive endoN, as described above. The samples were analyzed by Western blotting against histone H1. A histone mixture from calf thymus was used as a positive control (Figure 4A). The molecular weight of histone H1, without any posttranslational modification, was calculated as 21 kDa. Our control visualized modified and degraded variants of histone H1 in calf thymus. Proteolysis, degradation and clipping of histones play important roles during biological activities like epigenetics, normal cell growth and immunology (reviewed in [30]). In our polySia-samples from plasma, we detected a signal against histone H1 with a mass of ~25 kDa and further signals between 40–80 kDa. The band with a lower mass weight corresponds to the migration behavior of histone H1 with few or no modifications. The signals at the higher mass weight were probably ubiquitylated histone H1, which might be remnants of death cells. Histone H1 was described as the main target for this modification after DNA double-strand breaks [31]. Thus, the results suggest that polySia-histone complexes exist in donor plasma samples.

### 2.5. PolySia in Plasma Can Inhibit the Cytotoxicity of Histones

Based on the results, we wanted to examine if polySia from plasma can reduce histone-mediated cytotoxicity. For this purpose, cells were treated with histones and polySia was added to a parallel sample setting and isolated by inactive endoN-coated magnetic beads from plasma. As shown in Figure 4B, the histone-mediated cytotoxicity was significantly reduced by polySia from plasma. To determine the direct impact of polySia, samples were pretreated with endoN to degrade polySia. The preceding degradation of polySia by endoN prevented the cytoprotective effect. This result indicates that the observed effect was mediated by polySia. Remarkably, polySia is still able to reduce histone-mediated cytotoxicity, although complexes with histones from plasma already exist. Thus, in donor samples, the full capacity of polySia to bind and inactivate extracellular histones was not completely exhausted.

### 2.6. Several Branches of Vertebrates Have PolySia in Plasma

**Figure 5:**
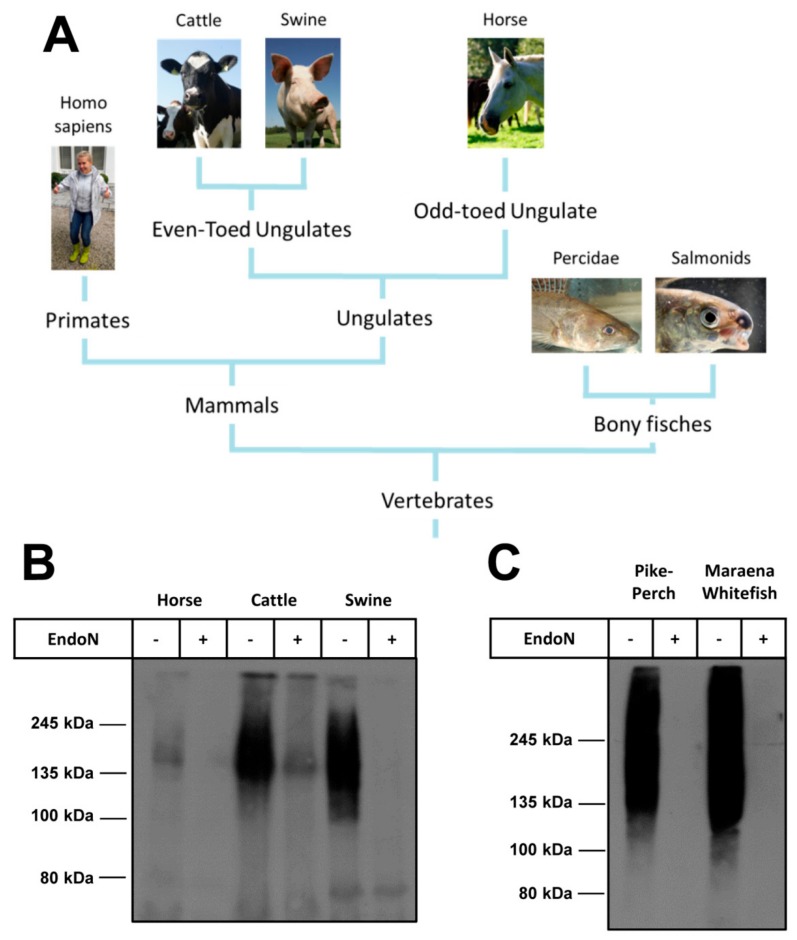
Evolutionary relationship of species with polySia in plasma. The presence of polySia in plasma was tested in several branches of vertebrates. (**A**) The relationships of examined species are shown in a phylogenetic tree. PolySia occurs in plasma of different vertebrate branches; (**B**,**C**) 1 µL plasma of different individual was used for western blotting. PolySia was detected with mAb 735. The samples were pretreated with endoN to confirm a specific signal; (**B**) The tested plasma was from mammals’ horse, cattle and swine; and (**C**) Plasma originates from fishes (pike-perch and maraena whitefish).

Since the release of NETs occurs not only in human but also in other vertebrates, we tested plasma samples of animals belonging to other vertebrates (Figure 5A). In a first set of experiments, plasma samples of mammals belonging to the “classical” farm animals were investigated for polySia by Western blotting as outlined above. As shown in Figure 5B, plasma obtained from horse, cattle and swine were polySia positive. Thus, plasma of other mammalian families also contains polySia.

Interestingly, NETosis seems to be a very common mechanism, since the beneficial suicide of neutrophils was also described in fish [32]. We tested the plasma of two different bony fishes: pike-perch (*Sander lucioperca*) belonging to the family of percidae and maraena whitefish (*Coregonus maraena*) belonging to the salmonids (Figure 5C). In line with mammals, fish plasmas showed a typical smear for polySia, when Western blot analyses were performed.

The obtained results strongly suggest that polySia is a physiological plasma component in vertebrates. Based on the additional observation that polySia of human plasma samples and the presence of polySia in plasmas of animals belonging to different branches of vertebrates we propose that polySia might be a part of a natural buffer system for extracellular histones. However, also other roles of polySia might be possible like a modulation of coagulation. A very recent review discusses the possibility of NETs to trigger thrombosis during sterile inflammation (e.g., cancer) as well as infections by pathogens and the role of platelets to modulate NET-formation [33]. Since histones can mediate platelet activation, polySia might be able to modulate this activation.

## 3. Materials and Methods

### 3.1. Materials

PolySia-specific monoclonal antibody (mAb) 735 and inactive and active endoN were provided by Martina Mühlenhoff (MHH Hannover, Germany) [34,35]. Equine blood samples were collected from three four years old Mecklenburger warmblood mares in heat via jugular vein puncture (permission: Az: 7221.3-2.3.1-004/10) and three bovine blood samples were provided from 15-month-old German Holstein heifers by puncturing the coccygeal vessels (permission: Az: 7221.3-2.3-003/13). All blood samples were filled in blood collection tubes for plasma preparation containing 1.6 mg EDTA-K/mL blood (S-Monovette^®^ EDTA 9 mL, Sarstedt, Nuembrecht, Germany). Pig plasma samples were obtained from animals killed by electronarcosis and exsanguination in the Institute’s experimental slaughterhouse (permission: AZ-7221.3-1-053-15). Trunk blood (50 mL) was collected in EDTA-containing tubes (1 mL 0.5 M EDTA) and stored on ice. Blood from maraena whitefish (300.6 g ± 68.5 g) and pike-perch (13.0 g ± 0.3 g), respectively, was sampled from the caudal vein of each individual using a 5 mL plastic syringe filled with 500 µL 0.5 M EDTA (pH 8.0) solution. All plasma samples were centrifuged (3500× *g*, 10 min, 4 °C) directly after collection and stored at −20 °C until analysis. Human plasma experiments were performed with pooled normal human plasma (Innovative Research, Novi, MI, USA). The study was conducted according to the Declaration of Helsinki. All reagents used were of analytical grade.

### 3.2. Enrichment of Polysialylated EVs and Western Blot Analysis

In order to isolate polySia from plasma, inactivated endoN was loaded onto Tosylactivated Dynabeads^®^ M-280 (Life Technologies, Oslo, Norway), as described previously [21]. EVs were enriched using a Total Exosome Isolation (from plasma) Kit and/or the Exosome-Human CD63 Isolation/Detection Kit (both from invitrogen™ life technologies, Oslo, Norway). Purified polysialylated carriers and/or EVs were separated by 10% SDS-PAGE under reducing conditions, followed by the transfer of samples onto a PVDF membrane. Plasma samples of different species were pretreated with 1× RIPA-Buffer for 30 min and a subsequent incubation with active endoN for 1 h at 37 °C. The plasma samples were analyzed by 7% SDS-PAGE and transferred also onto PVDF membrane. For the detection of CD63, an antibody from System Biosciences (Palo Alto, CA, USA) was used. Immunostaining against polySia was done with mAb 735 (1 µg/mL). Horseradish peroxidase (HRP)-conjugated secondary antibodies (Dako, Hamburg, Germany) were applied for visualization. Finally, a chemiluminescence signal was developed with light-sensitive Amersham Hyperfilm™ ECL (GE Healthcare, Buckinghamshire, UK) plus development solution.

For the determination of polySia binding partners, plasma or EVs were purified via inactive endoN coupled to Dynabeads, as described above, frozen and dried. These samples were resolved in lysis buffer (20 mM tris, 0.5% triton X-100). A histone mixture from calf thymus was used as an additional control (Sigma-Aldrich, Steinheim, Germany). The samples and controls were separated on a 15% Tricine-SDS-Page with 6 M urea, as described by Schägger and Jagow [36], then transferred to a PVDF membrane. For the detection of histone H1, an anti-histone H1 antibody (Merck Millipore, Billerica, MA, USA), in combination with an anti-mouse-HRP-conjugated antibody (Jackson ImmunoResearch, West Grove, PA, USA), was used.

### 3.3. Transmission Electron Microscopy

EVs-containing pellets were fixed with 1.5% glutaraldehyde and 1.5% formaldehyde (freshly made from paraformaldehyde) in 0.15 M HEPES buffer. For epoxy resin embedding, pellets were postfixed in 1% osmium tetroxide in aqua bidest, stained in half-saturated watery uranyl acetate, dehydrated in an ascending ethanol series and finally embedded in Agar 100 (Agar scientific Ltd., Stansted, UK). Ultrathin sections were cut using an ultramicrotome and examined by TEM (Zeiss EM 902, Oberkochen, Germany). Digital images were captured with a slow-scan 2K CCD camera (TRS, Tröndle, Moorenweis, Germany).

### 3.4. Determination of Chain Length Distribution by HPLC

The sample’s degree of polymerization (DP) was analyzed by “mild” DMB labeling via anion exchange chromatography, as described previously [37,38,39,40]. The DMB reaction buffer contained 9 mM sodium hydrosulfite, 0.5 M β-mercaptoethanol, 20 mM trifluoroacetic acid (TFA) and 1.35 M DMB (Dojindo, Kumamoto, Japan). The labeling happened overnight at 11 °C and was stopped by the addition of 1 M NaOH [40]. The samples were separated on a DNAPac PAc-100 column (4 × 250 mm; 13 µm; Thermo, Idstein, Germany) by HPLC (Smartline System, Knauer, Berlin, Germany) [41]. For eluents, we used MilliQ water (E1) and 2 M ammonium acetate buffer (E2) at a flow rate of 1 mL/min using the following gradient: 0 min = 0% E2; 5 min = 0% E2; 15 min = 8% E2; 20min = 11% E2; 35 min = 14% E2; 55 min = 16% E2; 100 min = 20% E2; 130 min = 23% E2; and 131 min = 100% E2. The labeled chains of polySia were detected by a fluorescence detector (372 nm for excitation and 456 nm for emission).

### 3.5. Cell Culture Experiments

For cell culture experiments, polySia was purified as described above. 5B8 cells were cultivated and treated as described in detail earlier [39]. For the experiments, 30,000 cells per well were seeded on a 96-well plate. In addition to untreated cells, cells were incubated with histones (40 µg/mL; Sigma Aldrich) at 37 °C and 5% CO_2_ for 1 h 45 min. In order to test the impact of polySia originating from plasma, native polySia- or endoN-treated polySia was added. The polySia of each well corresponded to the amount of polySia in 125 µL plasma. The cytotoxicity was determined by a lactate dehydrogenase (LDH) cytotoxicity assay (BioVision, Milpitas, CA, USA).

## 4. Conclusions

We observed that the chain length of polySia originating from plasma is long enough to be able to bind to histones and be cytoprotective against these toxic proteins. Interestingly, parts of polySia from plasma are already bound to histone H1. Despite this, polySia can still reduce further histone-mediated cytotoxicity and protect cells against it. Taken together, the results suggest that polySia might be a conserved natural endogenous counterpart to extracellular histones in the blood of vertebrates.

## Abbreviations

DMB	4,5-methylene dioxybenzene
DP	degree of polymerization
endoN	endoneuraminidase
EVs	extracellular vesicles
LDH	lactate dehydrogenase
mAb	monoclonal antibody
NETs	neutrophil extracellular traps
PolySia	polysialic acid
TEM	transmission electron microscopy
